# The Teeth from a Medico-legal Aspect

**Published:** 1887-05

**Authors:** 


**The Teeth from a Medico-legal Aspect.**—The identification of dead bodies and criminals is sometimes a matter of much perplexity. For instance, the features of a dead body may be distorted or destroyed; the clothes changed or unrecognizable; and no ordinary circumstances left to make identification clear. Some such a case occurred in Michigan. A man was found in a lake murdered. As the coroner was about dismissing the case as "undentified," the neighboring dentist had the curiosity to look into the mouth. In a moment he said: "I have a chart of that mouth in my office," and though he could not then remember the name, he soon found it by referring to his chart book. It resulted in tracing the murderer.—*Medical News.*

